# Identifying 8-mRNAsi Based Signature for Predicting Survival in Patients With Head and Neck Squamous Cell Carcinoma via Machine Learning

**DOI:** 10.3389/fgene.2020.566159

**Published:** 2020-10-29

**Authors:** Yuxi Tian, Juncheng Wang, Chao Qin, Gangcai Zhu, Xuan Chen, Zhixiang Chen, Yuexiang Qin, Ming Wei, Zhexuan Li, Xin Zhang, Yunxia Lv, Gengming Cai

**Affiliations:** ^1^Department of Oncology, Xiangya Hospital, Central South University, Changsha, China; ^2^Department of Otolaryngology Head and Neck Surgery, Xiangya Hospital, Central South University, Changsha, China; ^3^Department of Neurosurgery, The First People’s Hospital of Changde City, Changde, China; ^4^Department of Otolaryngology Head and Neck Surgery, The Second Xiangya Hospital, Central South University, Changsha, China; ^5^Department of Stomatology, Changzheng Hospital, Second Military Medical University, Shanghai, China; ^6^Hainan General Hospital, Hainan Affiliated Hospital of Hainan Medical University, Haikou, China; ^7^Department of Health Management, The Third Xiangya Hospital, Central South University, Changsha, China; ^8^Department of Thyroid Surgery, The Second Affiliated Hospital of Nanchang University, Nanchang, China; ^9^Department of Otolaryngology Head and Neck Surgery, First Affiliated Hospital of Quanzhou, Fujian Medical University, Quanzhou, China

**Keywords:** cancer cell stemness indices, head and neck squamous cell carcinomas, The Cancer Genome Atlas, weighted gene co-expression network analysis, predictive models

## Abstract

Cancer stem cells (CSCs) have been characterized by several exclusive features that include differentiation, self-renew, and homeostatic control, which allows tumor maintenance and spread. Recurrence and therapeutic resistance of head and neck squamous cell carcinomas (HNSCC) have been identified to be attributed to CSCs. However, the biomarkers led to the development of HNSCC stem cells remain less defined. In this study, we quantified cancer stemness by mRNA expression-based stemness index (mRNAsi), and found that mRNAsi indices were higher in HNSCC tissues than that in normal tissue. A significantly higher mRNAsi was observed in HPV positive patients than HPV negative patients, as well as in male patients than in female patients. The 8-mRNAsi signature was identified from the genes in two modules which were mostly related to mRNAsi screened by weighted gene co-expression network analysis. In this prognostic signatures, high expression of *RGS16, LYVE1, hnRNPC, ANP32A*, and *AIMP1* focus in promoting cell proliferation and tumor progression. While *ZNF66, PIK3R3*, and *MAP2K7* are associated with a low risk of death. The riskscore of eight signatures have a powerful capacity for 1-, 3-, 5-year of overall survival prediction (5-year AUC 0.77, 95% CI 0.69–0.85). These findings based on stemness indices may provide a novel understanding of target therapy for suppressing HNSCC stem cells.

## Introduction

Head and neck cancers are a collection of malignancies that arise from the upper aerodigestive tract, salivary glands and thyroid (Cramer et al., 2019). Head and neck squamous cell carcinomas (HNSCC) account for 90% of head and neck cancers and are mainly derived from the oral cavity, oropharynx, hypopharynx, and larynx (Wyss et al., 2013). The main reasons associated with their occurrence are tobacco and alcohol use, however, increased HNSCC cases with human papillomavirus (HPV) have highlighted the role of high-risk HPV in the pathology of HNSCC (Chaturvedi et al., 2011). Worldwide, around 430,000 patients die due to its high mortality annually, where its 5-year survival rate is about 40–50%, though patients with the advanced disease only have a 34.9% survival rate (Leemans et al., 2011). Hence, it is critical to explore the mechanism regarding this malignancy, which may aid in diagnosing early HNSCC and predicting clinical outcomes.

Stem cells are known to be a cell subset having the ability to self-renew and differentiate, which has been found in most human tissues (Blanpain et al., 2004). Due to strides in cancer research, cancer cells are generally considered to have the propensity to initiate, spread and metastasize. Several studies based on multiple tumors showed that a small subpopulation of undifferentiated cells that strikingly resemble stem cells within the tumor could trigger cancers. Therefore, these cells were aptly named cancer stem cells (CSCs; Reya et al., 2001). Cancer stem cells are present in bulk tumors of HNSCC and gave rise to new tumors in immunodeficient mice (Prince et al., 2007; Okamoto et al., 2009), which may elucidate how residual stem cells cause tumor recurrence and regrowth in patients following treatment. To further clarify CSCs, researchers fused artificial intelligence and deep learning methods further to explore the features of stem cells in tumors. Malta et al. (2018) generated stemness indices for evaluating the degree of oncogenic dedifferentiation using a one-class logistic regression machine learning algorithm (OCLR), which may define signatures to quantify stemness. Accordingly, they extracted transcriptomic and epigenetic feature sets from non-transformed pluripotent stem cells and their differentiated progeny, eventually obtaining the two stemness indices, mDNAsi and mRNA expression-based stemness index (mRNAsi).

This study attempts to generate the stem cell-associated indices by taking advantage of both the Progenitor Cell Biology Consortium (PCBC) and The Cancer Genome Atlas (TCGA) databases, which analyzed and quantified cancer stemness in the HNSCC cohort and acquired their mRNAsi scores. Using weighted gene co-expression network analysis (WGCNA), gene modules were constructed that are closely related to the stem index. Eight mRNAsi based signatures were selected from two of these gene modules, and a risk model based on eight mRNAsi signatures was conducted to predict the prognostic risk in HNSCC patients. Finally, a functional analysis was carried out to determine the molecular mechanism’s stemness regarding the prognosis of HNSCC patients.

## Materials and Methods

### Data Collection and Pre-processing

The CSC samples were downloaded from the PCBC R package synapser (v 0.6.61). Moreover, the raw data of gene expression and related clinical information of HNSCC patients were downloaded from the TCGA website, which included 546 RNA-Seq expression data. Additionally, 97 cases of GSE41613 data were downloaded from the Gene Expression Omnibus (GEO) website. The RNA-Seq data from TCGA-HNSCC were pre-processed as follows. Samples with expression profile information were retained, changing the Ensemble ID to Gene Symbol, while only leaving protein-coding genes. Next, the expression data of primary solid tumors and solid normal tissue samples were left. Afterward, the expression of multiple genes was chosen as the median. Finally, the overall survival (OS) data used for the survival analysis removed samples with a survival time of less than 30 days. GSE41613 data was also pre-processed, and the samples kept their expression profile information. Moreover, the unit of survival information of the sample was converted to days, and the probe was changed to the Gene Symbol. The probes which were related to several genes were deleted, and the expression of multiple genes was chosen as the median. As the TCGA data, the OS data used for the survival analysis removed samples with a survival time less than 30 days. All data from these two databases after pre-processing are shown in Table 1.

**TABLE 1:** Clinical information of TCGA-HNSCC and GSE41613.

**Clinical features**	**TCGA-STAD**	**GSE41613**
**Type**		
Normal	44	0
Tumor	500	97
**OS**		
0	280	46
1	211	50
**OS time (mean)**		
0	1047.261	1997.23
1	767.1185	730.65
**T Stage**		
T1	34	
T2	143	
T3	132	
T4	180	
TX	11	
**N Stage**		
N0	241	
N1	81	
N2	152	
N3	7	
NX	19	
**M Stage**		
M0	475	
M1	5	
MX	20	
**Stage**		
I	25	
II	81	
III	90	
IV	304	
**Grade**		
G1	61	
G2	299	
G3	119	
G4	2	
GX	19	
**Gender**		
Male	367	
Female	133	
**Age**		
≤60	244	
>60	255	
Unknown	1	
**Alcohol**		
Yes	332	
No	157	
Unknown	11	
**HPV Status**		
Negative	64	
Positive	19	
Unknown	417	
**Tobacco**		
1	111	
2	170	
3	72	
4	135	

### CSCs-Related Clinical Characteristics of HNSCC

The expression data of pluripotent stem cells (ESC and iPSC) from the PCBC database were analyzed, and the OCLR algorithm was utilized to predict mRNAsi. The Kruskal-Wallis test then compared the mRNAsi of normal tissue and tumor tissue or different clinical characteristics.

### Weighted Gene Co-expression Network Analysis

#### Module Establishment

The WGCNA co-expression algorithm was utilized to acquire the co-expressed genes and co-expression modules according to the expression profiles of these genes. According to the 500 HNSCC expression data from the TCGA database, the expression profiles of the protein-coding genes were extracted. A co-expression network was constructed using WGCNA in the R package based on the TCGA datasets. A Pearson correlation matrix was built to calculate the distance of each gene.

In this study, a soft threshold of nine was selected to screen the co-expression modules. To ensure the constructed co-expression network approached the scale-free distribution, β = 9 was chosen. Next, the expression matrix was changed to the adjacency matrix, after which the adjacency matrix was converted into a topological overlap matrix (TOM). Average linkage hierarchical clustering was used to cluster genes based on TOM, and the minimum genome number of the gene dendrogram was 40.

#### Identifying mRNAsi Modules

After determining the genetic modules, the module eigengenes of each module, in turn, was calculated, and the modules were then clustered, resulting in 20 differently related modules. The relationship between each module and different clinical characteristics was also analyzed. The most positive correlation was with the blue module, while the most negative correlation was with the yellow module.

### Functional Annotation: Gene Ontology and Kyoto Encyclopedia of Genes and Genomes Analyses

The WebGestaltR (v0.4.2) R package was adopted for the Kyoto Encyclopedia of Genes and Genomes (KEGG) pathway enrichment analysis and Gene Ontology (GO) functional annotation to investigate the biological functions of key modules and genes. In our study, we identify over-represented GO terms in three different categories: biological processes, molecular function and cellular component, and over-represented KEGG pathway terms. Furthermore, FDR < 0.05 was considered to be statistically significant.

### Construction and Analysis of the Risk Prognosis Model

The 491 TCGA samples were random as a 0.5:0.5 ratio divided into the training and test sets as previously described (Wang et al., 2020). Then, using the training set samples, the genes were further identified using a univariate Cox regression analysis of the survival coxph function package in the R language, where *p* < 0.01 was used as the threshold to optimize the data. Least absolute shrinkage and selection operator (Lasso) regression analysis was then used to reduce the number of genes, resulting in 17 genes. Next, the Akaike information criterion (AIC) was utilized to optimize the data, and a total of eight genes were finally identified for further use. The corresponding eight genes were used to build a prognostic risk score model.

The formula of the risk score model is described as:

$$R\,i\,s\,k\,S\,c\,o\,r\,e=0.20799^{\times }\,R\,G\,S\,16+0.2492^{\times }\,L\,Y\,V\,E\,1-0.8828^{\times }$$

$$M\,A\,P\,2\,K\,7-0.2654^{\times }\,P\,I\,K\,3\,R\,3-0.5666^{\times }\,Z\,N\,F\,66$$

$$+0.6486^{\times }\,h\,n\,R\,N\,P\,C+0.7821^{\times }\,A\,N\,P\,32\,A+0.5284^{\times }\,A\,I\,M\,P\,1$$

We used TCGA training set to test whether the gene markers were independent prognostic factors, and multivariate Cox regression analysis was used. Receiver operating characteristic (ROC) curve was depicted using the timeROC package in R. Samples in H (High) set had a significantly higher score compared to those in the L (Low) set, where “0” was used to divide the two sets. A Kaplan–Meier (KM) curve was drawn. Significance was defined as *P* < 0.05.

### Module and Clinical Trait Association Prognosis Analysis

The relationship between different clinical traits and OS time survival curves were plotted from the KM estimates. For the 8-mRNAsi based signature associations, some groups were clearly distinct to high or low expression groups.

### Gene Set Enrichment Analysis and Gene Set Variation Analysis (GSVA)

The R package was employed to perform the gene set enrichment analysis (GSEA) analysis of the key genes. Meanwhile, the “gene set variation analysis (GSVA)” R package was used to find the most associated pathways with the 8-mRNAsi based signature. Based on the different functions according to the score of each sample, the correlation between these functions and risk was further calculated, and the most associated pathways were identified.

### Cell Culture

Human HNSCC cell lines FaDu, JHU011 and HN8 were kindly provided by the Xiangya Hospital of Central South University. FaDu cell was cultured in MEM medium (Sigma, MO, United States), JHU011cell was cultured in RPMI-1640 and HN8 cell was cultured in DMEM medium (Sigma, MO, United States). All the medium were supplemented with 10% FBS and 1% penicillin/streptomycin, maintained on plastic plates and incubated at 37°C with 5% CO_2_.

### RT-qPCR Assay

According to the manufacturer’s protocol, total RNA of cells was extracted using TRIzol (Life Technologies, Carlsbad, CA, United States). After cDNA synthesis (All-in-One First-Strand cDNA Synthesis kit, GeneCopoeia Inc, Santa Cruz, CA, United States), the quantitative real-time polymerase chain reaction (qPCR) experiment was carried out using All-in-One qPCR Mix (GeneCopoeia Inc, United States) on ABI 7500HT System (Applied Biosystems, Foster City, CA, United States) using primers were described as Supplementary Table 1. The PCR detailed reaction conditions were as follows: 95°C for 5 min followed by 40 cycles of 95°C for 10 s, 60°C for 20 s and 72°C for 20 s. GAPDH was used as the internal control in this study. The relative expression of target genes was controlled to GAPDH and 2^–ΔΔC*T*^ method was calculated to evaluate relative mRNA levels. All the experiments were run in triplicate.

## Results

### Relationship Between mRNAsi and Clinical Characteristics in Head and Neck Cancer

**FIGURE 1:**
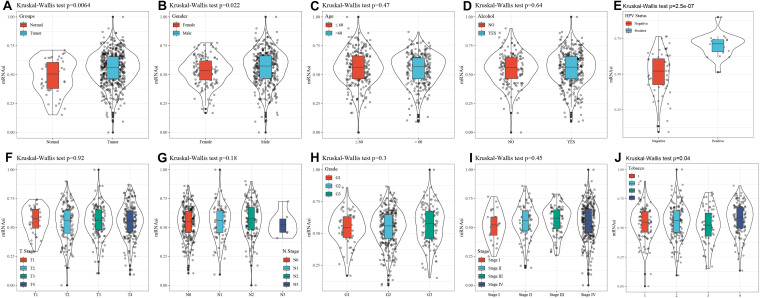
Correlation between mRNAsi and clinical characteristics in HNSCC. **(A)** The different expressions of mRNAsi between normal and tumor samples. **(B)** The different expressions of mRNAsi between gender-specific samples. **(C)** The different expressions of mRNAsi between different age samples. **(D)** The different expressions of mRNAsi between drinking alcohol status samples. **(E)** The different expressions of mRNAsi between different HPV status samples. **(F)** The different expressions of mRNAsi between different T staging. **(G)** The different expressions of mRNAsi between different N staging samples. **(H)** The different expressions of mRNAsi between different Grade grading samples. **(I)** The different expressions of mRNAsi between different Stage staging samples. **(J)** The different expressions of mRNAsi between smoking status samples.

mRNA expression-based stemness index is a particular stemness index, which is considered to be a biomarker in CSCs. 78 cases of expression data from pluripotent stem cells were downloaded from PCBC. Here, mRNAsi in HNSCC tissues was significantly higher than that of normal tissues (*p* = 0.0064) (Figure 1A). Moreover, to discover the correlation of mRNAsi with the corresponding clinical characteristics, the downloaded information contains the gender, age, disease stage, tumor stage classification, node stage classification, clinical grade, HPV status, smoking status, and alcohol status. The result of the Kruskal–Wallis test showed that male patients had a significantly higher mRNAsi than female patients (*p* = 0.022) (Figure 1B). Meanwhile, there was a difference in mRNAsi in the smoking status group (*p* = 0.04) (Figure 1J). And the result of Kruskal–Wallis test indicated that HPV positive patients had a significantly higher mRNAsi than HPV negative patients (*p* = 2.5e-07) (Figure 1E). In terms of age, alcohol status, tumor classification, node classification, and disease stage, no significant difference in the mRNAsi was present among the tumor tissues (Figure 1).

### WGCNA: Head and Neck Cancer Stem Cell Index and Gene Expression Analysis

**FIGURE 2:**
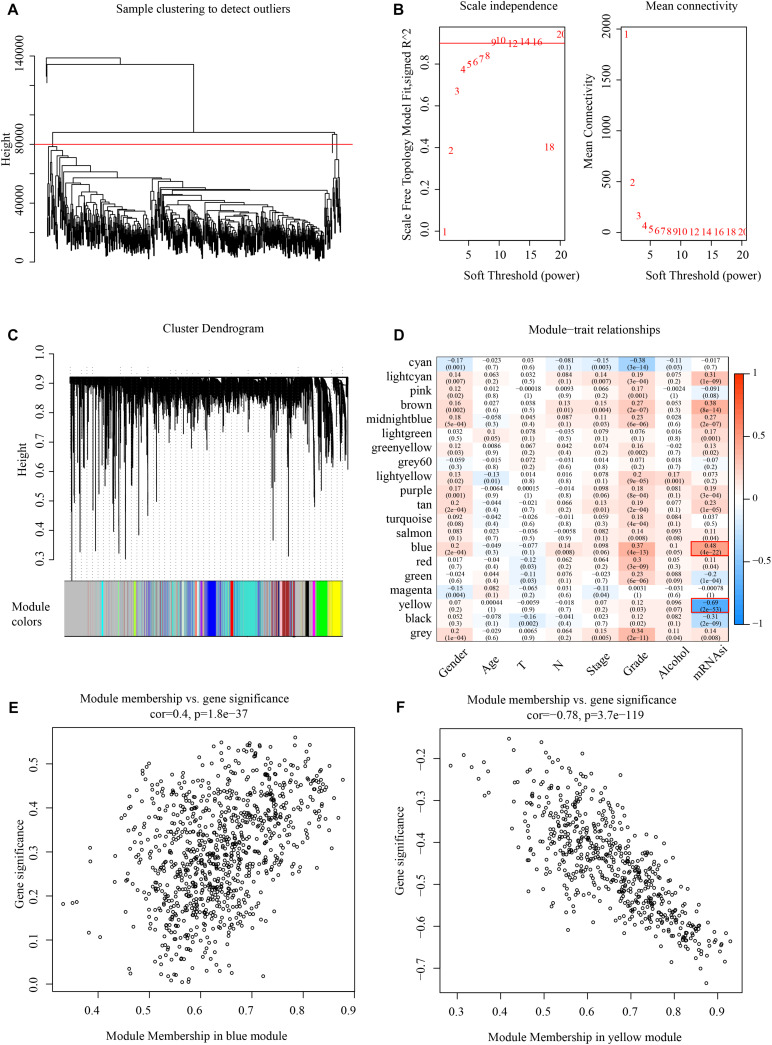
Identify mRNAsi basedgene modules in HNSCC. **(A)** Cluster analysis. **(B)** Analysis of network topology for various soft-thresholding powers. **(C)** Gene dendrogram and module colors. **(D)** Results of correlation between twenty modules and each clinical phenotype. **(E)** Correlation of blue modules and genes. **(F)** Correlation of yellow modules and genes.

Weighted gene co-expression network analysis analyzes the molecular interactions according to the co-expression network (Tian et al., 2018). Here, the expression profiles of protein-coding genes were selected according to 500 gene expression profiles of head and neck cancer from the TCGA database. Hierarchical clustering was then used to analyze sample clustering (Figure 2A). To this effect, β = 9 (Figure 2B) was chosen as a soft scale to ensure a scale-free network, culminating with 20 gene modules for further analysis (Figure 2C).

The correlation of mRNAsi with clinical factors like gender, age, TNM classification, and clinical stage was examined, as shown in Figure 2D, where the most significant positive correlation module with mRNAsi is the blue module, and the most negative correlation module with mRNAsi is the yellow module. And these two modules contain 1518 genes, and all the genes are shown in Supplementary Table 2. The module membership in the blue module was shown in Figure 2E and the module membership in the yellow module was shown in Figure 2F.

### Gene Modules Functional Annotation Analysis

This study employed GO and KEGG for the functional enrichment analysis of the blue and yellow modules. For the blue module, the study results show that all the top 10 significantly enriched factors with GO, Biological process (BP), Cellular component (CC), and KEGG pathways were obtained, as presented in Supplementary Figure 1. Notably, p53 signaling pathway, DNA replication and cell cycle are related to cancer, as we found in KEGG pathway analysis. Then for the yellow module, we can also get the results that the top 10 significantly enriched factors with GO, BP, CC, and KEGG pathways were presented in Supplementary Figure 2. Among all enriched KEGG pathways, the PI3K-Akt signaling pathway, MAPK signaling pathway and ECM–receptor interaction are related to cancer.

### Construct a Gene Prognostic Risk Model Based on mRNAsi

#### mRNAsi-Related Gene Prognostic Risk Models

The 491 samples were selected from TCGA and were randomly divided into training sets and test sets (Table 2). Additionally, 246 patients from the training set were used in the following survival analysis. According to the univariate Cox regression model and Lasso cox regression model, 17 genes were acquired for subsequent analysis. Afterward, AIC was used to optimize the data, and a total of eight genes were finally identified to analyze: *RGS16, LYVE1, MAP2K7, PIK3R3, ZNF66, hnRNPC, ANP32A*, and *AIMP1*.

**TABLE 2:** Clinical information statistics for TCGA train set and test set.

**Clinical Features**	**TCGA-train**	**TCGA-test**	***P***
**OS**			
0	146	134	0.3417
1	100	111	
**T Stage**			
T1	18	15	0.4751
T2	64	77	
T3	72	58	
T4	86	91	
TX	6	4	
**N Stage**			
N0	112	125	0.4721
N1	40	39	
N2	82	68	
N3	2	5	
NX	10	8	
**M Stage**			
M0	233	234	0.396
M1	4	1	
MX	9	10	
**Stage**			
I	10	15	0.3178
II	46	34	
III	41	49	
IV	149	147	
**Grade**			
G1	30	30	0.5258
G2	145	148	
G3	63	54	
G4	0	2	
GX	8	11	
**Gender**			
Male	180	181	0.9401
Female	66	64	
**Age**			
≤60	129	113	0.1904
>60	117	132	

**FIGURE 3:**
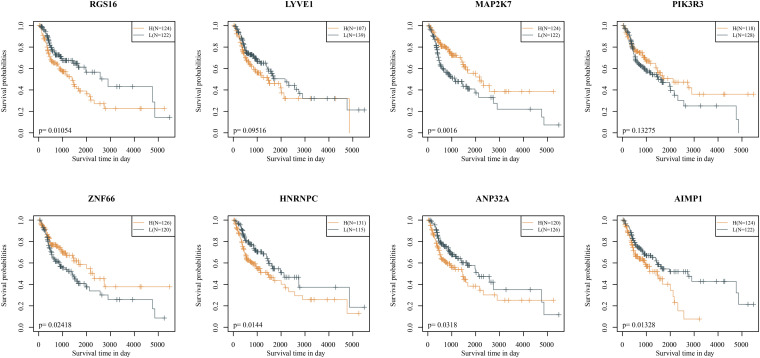
Kaplan–Meier curves of eight signatures (in the TCGA training set).

The KM curves showed that, except for LYVE1 and PIK3R3, the remaining six genes had significantly divided the samples from the training set into two groups, high risk groups and low risk groups (Figure 3).

**FIGURE 4:**
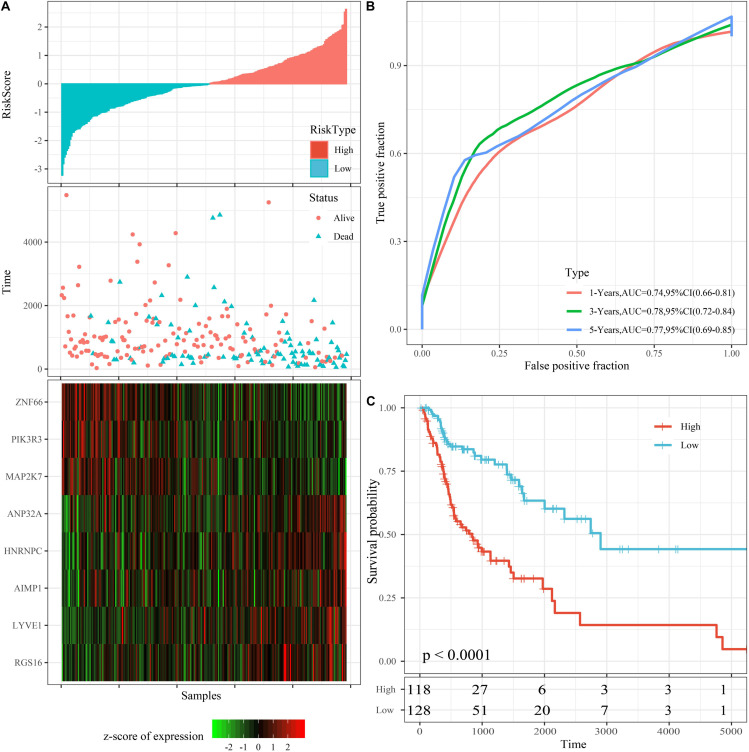
Performance of the 8-mRNAsi based signature model with TCGA training set. **(A)** Survival time, survival status and 8-genes expression of Riskscore in the training set. **(B)** ROC Curve and AUC of 8-gene signature Classification. **(C)** The KM survival curve distribution of 8-gene signature in the training set.

The riskscore of the training set was calculated according to the expression level of each sample, and the distribution of RS is shown in Figure 4A. The OS time of patients with high RS was found to be significantly lower than ones with low RS. *RGS16, LYVE1, hnRNPC, ANP32A*, and *AIMP1* with high expression represent risk factors. Moreover, *ZNF66, PIK3R3*, and *MAP2K7* attained the opposite result, making them protective factors. We further applied the timeROC package to analyze the prognosis of 1-, 3-, and 5-year survival rates. Accordingly, the model was found to exhibit that 1-year AUC 0.74, 95% CI 0.66–0.81, 3-year AUC = 0.78, 95% CI 0.72–0.84, and 5-year AUC 0.77 95% CI 0.69–0.85 (Figure 4B).

Additionally, riskscore was utilized to make the zscore, where all zscore samples greater than zero were included in the high risk group, while the rest of the samples smaller than zero were divided into the low risk group. Finally, 118 high risk samples and 128 low risk samples were obtained, the survival time between high and low risk samples was significantly (*p* < 0.0001; Figure 4C).

#### Risk Model Verification

To verify the robustness of the 8-mRNAsi based signature model, we calculated a riskscore in TCGA test set and an external dataset (GSE41613). Regarding the TCGA test dataset (Supplementary Figure 3), we found the same results as the training set was yielded for, where ROC analysis showed that the 5-year AUC was up to 0.70. The survival time between high and low risk samples was significantly different (*p* < 0.0001). For the GSE41613 database (Supplementary Figure 4), ROC analysis showed that the average 1-, 3-, and 5-year AUC for the 8-mRNAsi based signature was close to 0.67, 95%, the relationship between the expression of the eight genes and risk score is also consistent with the training set.

#### Risk Model and Analysis of Clinical Features of Prognosis

**FIGURE 5:**
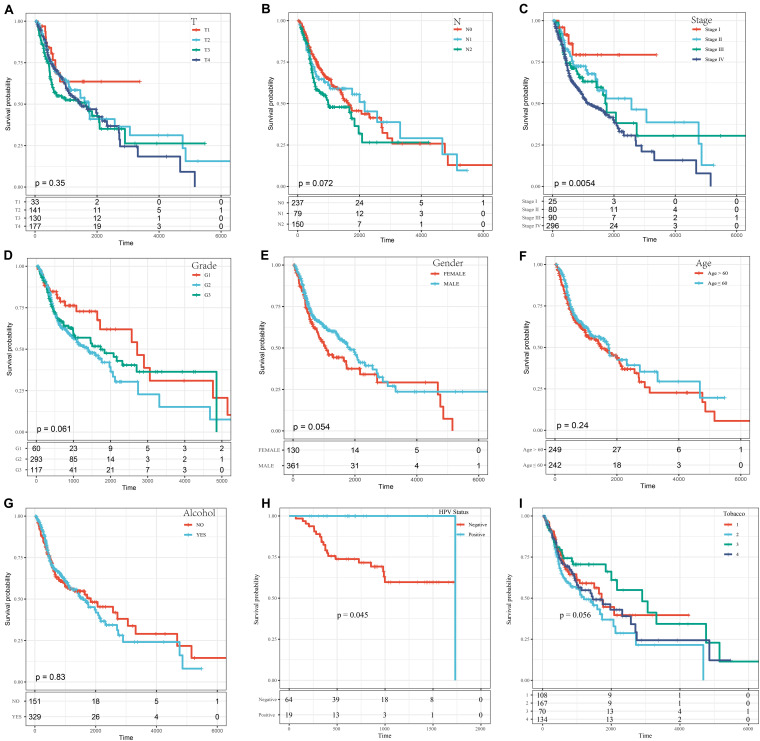
The KM curves of different clinical characteristics. **(A)** KM curves of different tumor classifications. **(B)** KM curves of different node classifications. **(C)** KM curves of different disease stages. **(D)** KM curves of different cancer grades. **(E)** KM curves of different genders. **(F)** KM curves of young (age ≤ 60) and elderly (age > 60) ages. **(G)** KM curves of different alcohol status. **(H)** KM curves of different HPV status. **(I)** KM curves of different smoking status.

A series of KM curves graphs were made to analyze the prognosis. As shown in Figure 5, patients with HNSCC were analyzed according to nine clinical features (tumor classification, Node classification, disease stage, grade, gender, age, alcohol status HPV status and smoking status). The meaning of the four different smoking status in Figure 5I was as follows: Lifelong Non-smoker (less than 100 cigarettes smoked in Lifetime) = Tabacco1; Current smoker (includes daily smokers and non-daily smokers or occasional smokers) = Tabacco2; Current reformed smoker for > 15 years (greater than 15 years) = Tabacco3; Current reformed smoker for ≤15 years (less than or equal to 15 years) = Tabacco4. The results showed that only the stage group and HPV status were related to OS time (*p* < 0.05) (Figures 5C,H), and the prognosis was worse with increasing disease stage and with HPV negative patients.

**FIGURE 6:**
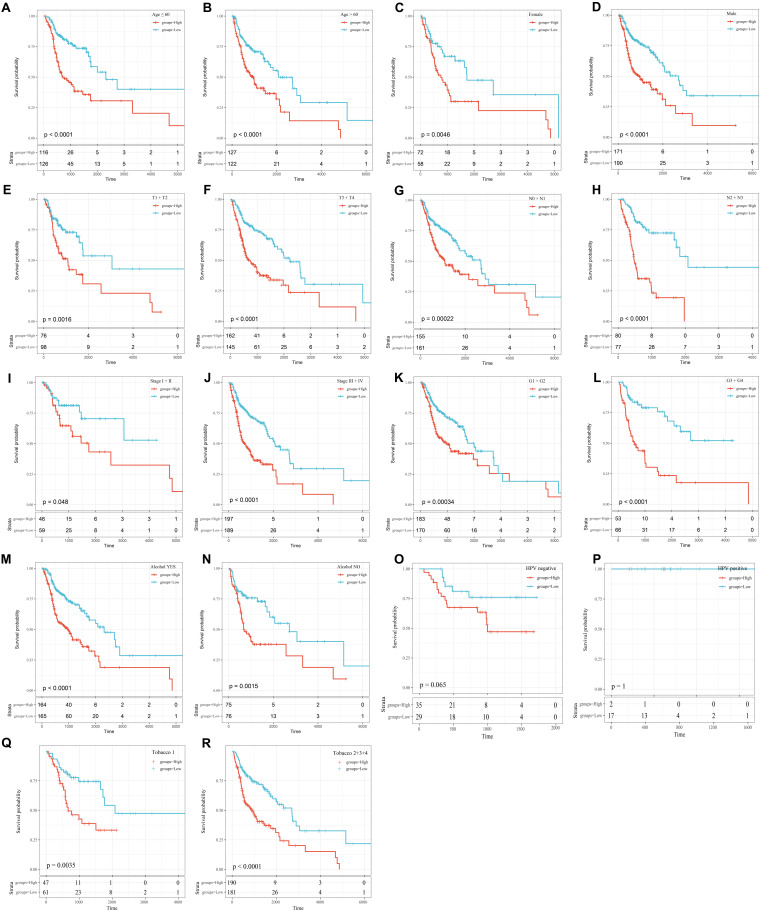
KM curves showing the OS of each subgroup of HNSCC patients with high or low riskscores. **(A)** KM curves of high and low risk samples in the young (age ≤ 60). **(B)** KM curves of high and low risk samples in the elderly (age > 60). **(C)** KM curves of Female samples. **(D)** KM curves of Male samples. **(E)** T1+T2 KM curves of high and low risk samples. **(F)** T3+T4 KM curves of high and low risk samples. **(G)** N0+N1 KM curves of high and low risk samples. **(H)** N2+N3 KM curves of high and low risk samples. **(I)** Stage I+II KM curves of high and low risk samples. **(J)** Stage III+IV KM curves of high and low risk samples. **(K)** G1+G2 KM curves of high and low risk samples. **(L)** G3+G4 KM curves of high and low risk samples. **(M)** KM curves of drinking samples. **(N)** KM curves of non-drinking samples. **(O)** KM curves of HPV negative samples. **(P)** KM curves of HPV positive samples. **(Q)** Tabacco1 KM curves of high and low risk samples. **(R)** Tabacco2+3+4 KM curves of high and low risk samples.

To further explore the influence of clinical features on the OS of the 8-mRNAsi based signature, all clinical features were stratified. Then, every stratified feature was divided into high-risk and low-risk groups. As shown in Figure 6, the 8-mRNAsi based signature acted as a risk factor for patients with different clinical characteristics.

We performed univariable and multivariable Cox regression analysis to evaluate the 8-mRNAsi based signature related HR, 95% CI of HR, *P*-value. Clinical characteristics, including alcohol status, age, tumor stage classification, node stage classification, pathological grade, disease stage, and riskscore, were systematically analyzed. Our results from the TCGA database showed that riskscore from either univariable (HR = 1.913, 95% CI 1.642-2.228, *p* = 2.0E-16) or multivariable Cox regression analysis(*HR* = 1.872, 95% CI 1.613-2.173, *p* = 2.0E-16) are significantly correlated to survival (Table 3). And the same result can be obtained in node stage classification and disease stage. In node stage classification group, univariable (HR = 1.205, 95% CI 1.045-1.389, *p* = 0.010) or multivariable Cox regression analysis (HR = 1.195, 95% CI 1.015-1.406, *p* = 0.032) are correlated to survival (Table 3). Meanwhile, in disease stage group, univariable (HR = 1.345, 95% CI 1.138-1.589, *p* = 5.0E-04) or multivariable Cox regression analysis (HR = 1.310, 95% CI 1.056-1.625, *p* = 0.014) are significantly correlated to survival (Table 3).

**TABLE 3:** Univariate and multivariate COX regression analyses of clinical factors.

**Variables**	**Univariable analysis**				**Multivariable analysis**			
	**HR**	**95% CI of HR**		***P***	**HR**	**95% CI of HR**		***P***
		**Lower**	**Upper**			**Lower**	**Upper**	
Age	1.017	1.005	1.030	0.007	1.022	1.008	1.035	0.001
Alcohol	1.025	0.792	1.326	0.850	0.927	0.710	1.212	0.581
T	1.099	0.962	1.256	0.164	0.907	0.776	1.059	0.216
N	**1.205**	1.045	1.389	**0.010**	**1.195**	1.015	1.406	**0.032**
Grade	1.096	0.915	1.313	0.318	1.051	0.867	1.274	0.612
Stage	**1.345**	1.138	1.589	**5.0E-04**	**1.310**	1.056	1.625	**0.014**
RiskScore	**1.913**	1.642	2.228	**2.0E-16**	**1.872**	1.613	2.173	**2.0E-16**

### Relationship Between Riskscore and Signaling Pathway

To analyze the KEGG functional enrichment score for each sample in the training set, GSVA was utilized in the R software package for the GSEA analysis.

**FIGURE 7:**
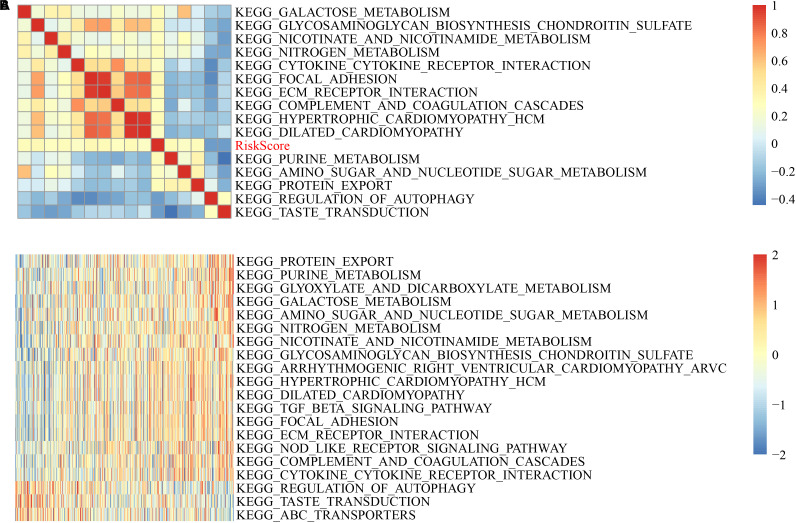
GSVA-derived clustering heatmaps of different pathways. **(A)** Clustering of correlation coefficients between KEGG pathways and RiskScore with a correlation greater than 0.25 with risk scores. **(B)** The correlation between the KEGG pathway and the risk score is greater than 0.25, and the ssGSEA score in each sample changes with the increase in risk score. The horizontal axis represents the sample, and the risk score increases in turn from left to right.

The scores were calculated from each sample with different functions to acquire the ssGSEA score of each function corresponding to each sample, where the relationship between functions and riskscore was further verified. The function with a correlation greater than 0.25 was selected, as shown in Figure 7A.

Here, 13 cases had a positively correlated with the sample risk score, while two had a negative correlation. The most related ten KEGG pathways were chosen and were clustered based on their enrichment score (Figure 7B). Accordingly, among all pathways, the riskscore rises as KEGG_COMPLEMENT_AND_COAGULATION_CASCADES, KEGG_NITROGEN_METABOLISM, and KEGG_TGF_BETA_ SIGNALING_PATHWAY rises, and for KEGG_REGULATION_ OF_AUTOPHAGY, KEGG_TASTE_TRANSDUCTION, KEGG _ABC_TRANSPORTERS, the riskscore decreases as they increase.

### Expression Level of Eight mRNAsi in HNSCC Cell Lines as Detected by a RT-qPCR Assay

**FIGURE 8:**
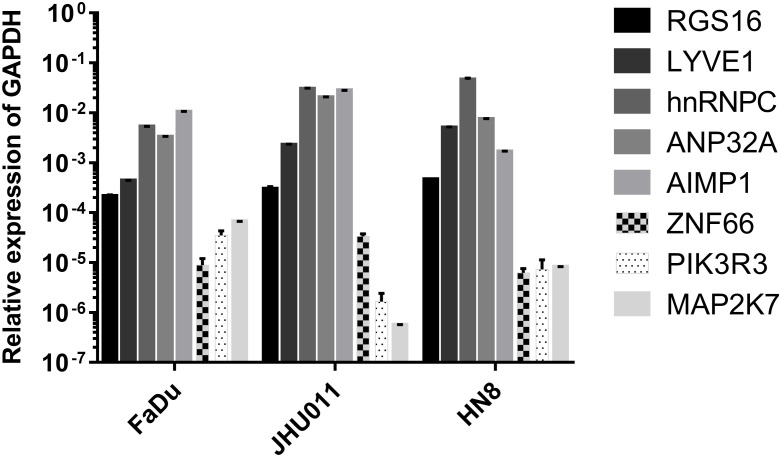
The transcriptional expression level of eight mRNAsi in HNSCC cell lines.

We tested the expression levels of eight mRNAsi in FaDu, JHU011, and HN8 cell lines by a RT-qPCR assay. The results showed that *RGS16*, *LYVE1, hnRNPC, ANP32A and A1MP1* were highly expressed in all cell lines. And *ZNF66, PIK3R3 and MAP2K7* were lowly expressed in three cell lines (Figure 8).

## Discussion

Many advanced therapeutic and diagnostic methods have been carried out in modern HNSCC treatment, though their effects remain inadequate as the oncologists anticipated. CSCs, due to their strong self-renewal ability, are thought to play an essential role associated with invasive potential, tumor growth and therapeutic resistance in response to the development of HNSCC (Peitzsch et al., 2019). Therefore, identifying therapeutic targets for CSCs would be significant in anti-cancer treatment. As a type of heterogeneous malignancy, a molecular analysis of HNSCC tissues demonstrates high intratumoral heterogeneity determined by clonal evolution of the CSCs populations (Yang et al., 2020). In the present study, the correlation of mRNAsi indices between normal tissues and HNSCC tissues were presented based on the OCLR machine-learning algorithm (Malta et al., 2018). In line with previous studies regarding other cancers (Malta et al., 2018; Lian et al., 2019), a significantly higher level of mRNAsi was observed in HNSCC tissues compared to that in normal tissues. By comparing the mRNAsi with the clinical characteristics, which revealed that mRNAsi had a significant rise in HPV positive patients, and that male patients had a higher mRNAsi indices than female patients. This result may suggest a potential correlation of HPV status with CSCs. One study of four HPV negative HNSCC cell lines were infected with HPV genome, which resulted in tumor cells have increased growth and self-renewal capacity (Lee et al., 2015). Zhang reported a study of six oropharyngeal HNSCC tumor specimens, where HPV positive tumors had a higher proportion of CSCs compared to HPV negative tumors in six specimens of HNSCC, which was attributed to p53 inactivation by HPV (Zhang et al., 2014). P53 is an essential target of HPV-E6/E7 proteins that bind to p53 resulting in the deregulation of p53 and causing a more proliferative state (Jin and Xu, 2015). Conversely, Tang determined that CSCs population are not affected by HPV in HNSCC (Tang et al., 2013). These databases suggested that the current understanding of the relationship between HPV status and CSCs is still weak. It will be interesting to perform additional research for the underlying mechanism.

By applying WGCNA, an important system in bioinformatics used to generate gene co-expression networks to detect gene modules and identify key genes (Langfelder and Horvath, 2008; Li et al., 2018), gene modules that were correlated with mRNAsi indices based on the gene expression profile of HNSCC samples were initially identified. In these modules, blue one had the most considerable positive correlation with mRNAsi indices, while yellow one had the opposite. Functional annotation could be beneficial in evaluating the impact of these gene modules on HNSCC. Regarding the blue module, major biological processes were involved in regulating the mitotic phase, organelle fission and negative regulation of the cell cycle. KEGG enrichment pathways in the blue module encompassed DNA replication, p53 signaling pathway and the cell cycle. KEGG enrichment pathways in the yellow module were mainly involved in ECM–receptor interactions, PI3K-Akt signaling pathways, and MAPK signaling pathways. These signaling pathways have been demonstrated to facilitate cell survival, self-renewal and apoptosis inhibition in many CSCs (Huang et al., 2017; Chen et al., 2020; Liao et al., 2020; Qin et al., 2020).

Key genes selected from mRNAsi correlated modules are currently employed in practice. Pan et al. (2019) screened 13 key genes based on mRNAsi associated gene modules in bladder cancer, which was shown to be related to stem cells. Pei et al. (2020) selected 12 mRNAsi based genes to be correlated with the survival of breast cancer patients. Zhang et al. (2020) showed 13 genes enriched in the cell cycle, which were increased due to the pathological stages of lung adenocarcinoma. These studies signified that there are inextricable links between key gene expressions and OS of patients. However, substantial evidence demonstrating that key genes may have predictive features in the clinical characteristics of cancer patients not yet elucidated. In the present study, 8-mRNAsi based signatures were established in predicting HNSCC. The riskscore was generated in samples of HNSCC based on expression patterns of these eight genes, which can serve as an independent predictor for OS in HNSCC patients (Table 2). The 8-mRNAsi based signature may also easily divide the HNSCC samples into high risk and low risk groups according to their various clinical characteristics required in the prognostic model for its potential use in clinical practice. Similar to our work, Cao and collaborators have evaluated the correlation between a three lncRNA signature patients OS with HNSCC by a log-rank test and univariable Cox regression. By OPLS-DA analysis and fold change selection, the three lncRNA signatures that can categorize patients into high and low risk groups have the highest predictive capacity. Comparatively, the same point is that univariable and multivariable Cox regression analysis were used to select the related genes in both studies. Otherwise, WGCNA and Lasso were performed in our study as the methods of dimensionality reduction for analyzing and selecting CSCs associated mRNA in HNSCC patients.

The 8-mRNAsi based prognostic model in our signatures includes *RGS16, LYVE1, hnRNPC, ANP32A, AIMP1, ZNF66, PIK3R3*, and *MAP2K7*, in which several genes have been reported to be linked with stemness features or be involved in cancer progression. *LYVE1*, lymphatic vessel endothelial hyaluronan receptor-1, has been identified as a biomarker of yolk sac endothelium and definitive hematopoietic stem and progenitor cells (HSPCs) by *Lyve1-Cre* labeling, where most HSPCs and erythro-myeloid progenitors were *Lyve1-Cre* lineage traced (Lee et al., 2016). *LYVE1* was thought to contribute to lymphangiogenesis in malignant tumors (Jackson et al., 2001). In the development of human embryonic stem cells, heterogeneous nuclear ribonucleoproteins (hnRNPs) has been identified as a critical regulator of physiologically relevant alternative cleavage and polyadenylation (APA) events that contribute to carcinogenesis by modulating the expression of genes that regulate cell proliferation and metastasis (Fischl et al., 2019). Silencing of hnRNPC can inhibit migratory and invasive activities by promoting miRNA-21 in brain tumor cells. Increased *hnRNPC* has been shown to contribute to cancer stemness and invasive potential in cancers (Park et al., 2012; Kleemann et al., 2018; Wu et al., 2018). However, the exact molecular function of *hnRNPC* needs to be explored in cancer stemness. *ANP32A*, acidic leucine-rich nuclear phosphoprotein-32A, expressed in normal tissue as well as multiple malignant tumors, several recent studies have indicated that *ANP32A* plays a significant role in cell proliferation, signal transduction, and other biological processes. Overexpression of *ANP32A* was associated with lymph node metastasis, which predicted poor survival in oral squamous cell carcinoma (OSCC) patients. Mechanical study indicates that *ANP32A* promotes tumor cell growth and may involve the inactivation of p38 and phosphorylation of Akt (Yan et al., 2017). *AIMP1* was identified as a cytokine that secretes in response to hypoxia and cytokine stimulation for involving cell proliferation regulation. A series of studies have shown that *AIMP1* can enhance wound healing by the mediation of fibroblast proliferation via ERK, and N-terminal domain (amino acids 6–46) of *AIMP1* was responsible for the stimulation of fibroblast proliferation (Park et al., 2005; Han et al., 2006). *AIMP1* peptide increased the expression of cyclin D1 and c-myc by stabilizing β-catenin through FGF receptor 2 (FGFR2)-mediated activation of Akt, which promotes the proliferation of bone marrow-derived mesenchymal stem cells (Kim et al., 2013). *ZNF66* is a member of the zinc finger transcription factor family which encounters many members and the gene coding for this protein is located on chromosome 19 in a fragile site region. Low mRNA expression of *ZNF66* is shown in head and neck cancers according to the TCGA dataset.^1^ However, the correlations between the features of CSCs and *ZNF66* is still unclear, and additional studies need to be performed to explore the role of *ZNF66* in the stemness of HNSCC. *PIK3R3* is one of the regulatory subunits of *PI3K* that positively correlates with cell proliferation signatures (Phillips et al., 2006). Furthermore, the expression of *PIK3R3* increased in neoplastic tissues compared to non neoplastic in patients with gastric cancer (Zhou et al., 2012). However, higher expression of *PIK3R3* has been reported in cancer patients with satisfactory colorectal cancer outcomes as it facilitated the apoptosis of cancer cells (Ibrahim et al., 2018). *MAP2K7* is a mitogen-activated protein kinase, encodes *MMK7* and acts through the *JNK* pathway for cell cycle arrest and suppression of epithelial cancers (Schramek et al., 2011).

The robustness of the 8-mRNAsi based signature was validated across the TCGA test set and an external data set (GSE41613). Although these findings have been validated in HNSCC cell lines, further validation is still required in matched tissues of HNSCC patients. Additionally, the molecular process and signaling pathway obtained across the TCGA cases alone are inadequate and need to be confirmed through further investigation.

## Conclusion

In our eight mRNAsi based signature, high expression of *RGS16, LYVE1, hnRNPC, ANP32A*, and *AIMP1* are correlated with a high risk of death as these genes focus in promoting cell proliferation and tumor progression, similar to stem cells. Regarding the other three genes, higher expression levels of *ZNF66, PIK3R3*, and *MAP2K7* are associated with a low risk of death. Interestingly, the molecular functions of these genes mainly concentrate on repressing the cell cycle and fostering apoptosis. Moreover, the present GSEA analysis discovered the mechanism regarding the KEGG pathway, which underlies the riskscore of the 8-mRNAsi based signature. Accordingly, to the best of our knowledge, all genes in the proposed mRNAsi based prognostic model have not been studied in HNSCC and may offer insight into the development of targeted therapies for HNSCC.

## Data Availability Statement

All datasets presented in this study are included in the article/Supplementary Material.

## Author Contributions

YT designed the study and analyzed the data. JW and YT carried out the manuscript. YT and CQ prepared the figures and tables. GZ, XC, ZC, YQ, MW, and ZL co-contributed to revising and polishing the manuscript. GC, XZ, and YL collated the data and supervised the study. All authors have read and approved the final submitted manuscript.

## Conflict of Interest

The authors declare that the research was conducted in the absence of any commercial or financial relationships that could be construed as a potential conflict of interest.

## Footnotes

1 https://www.proteinatlas.org

## Supplementary Material

Supplementary Figure 1 GO and KEGG pathway enrichment analysis in the blue module.

Supplementary Figure 2 GO and KEGG pathway enrichment analysis in the yellow module.

Supplementary Figure 3 Performance of the 8-mRNAsi based signature modelwith TCGA test dataset.

Supplementary Figure 4 Performance of the 8-mRNAsi based signature modelwithGSE41613 database.

Supplementary Table 1 The sequence of primers used for PCR in this study.

Supplementary Table 2 1518 genes information from yellow and blue modules.
