# Supplementary figures and images for: Identifying 8-mRNAsi Based Signature for Predicting Survival in Patients With Head and Neck Squamous Cell Carcinoma via Machine Learning
Source: Front Genet. 2020 Oct 29;11:566159. doi: 10.3389/fgene.2020.566159 (PMC7721480; doi:10.3389/fgene.2020.566159)

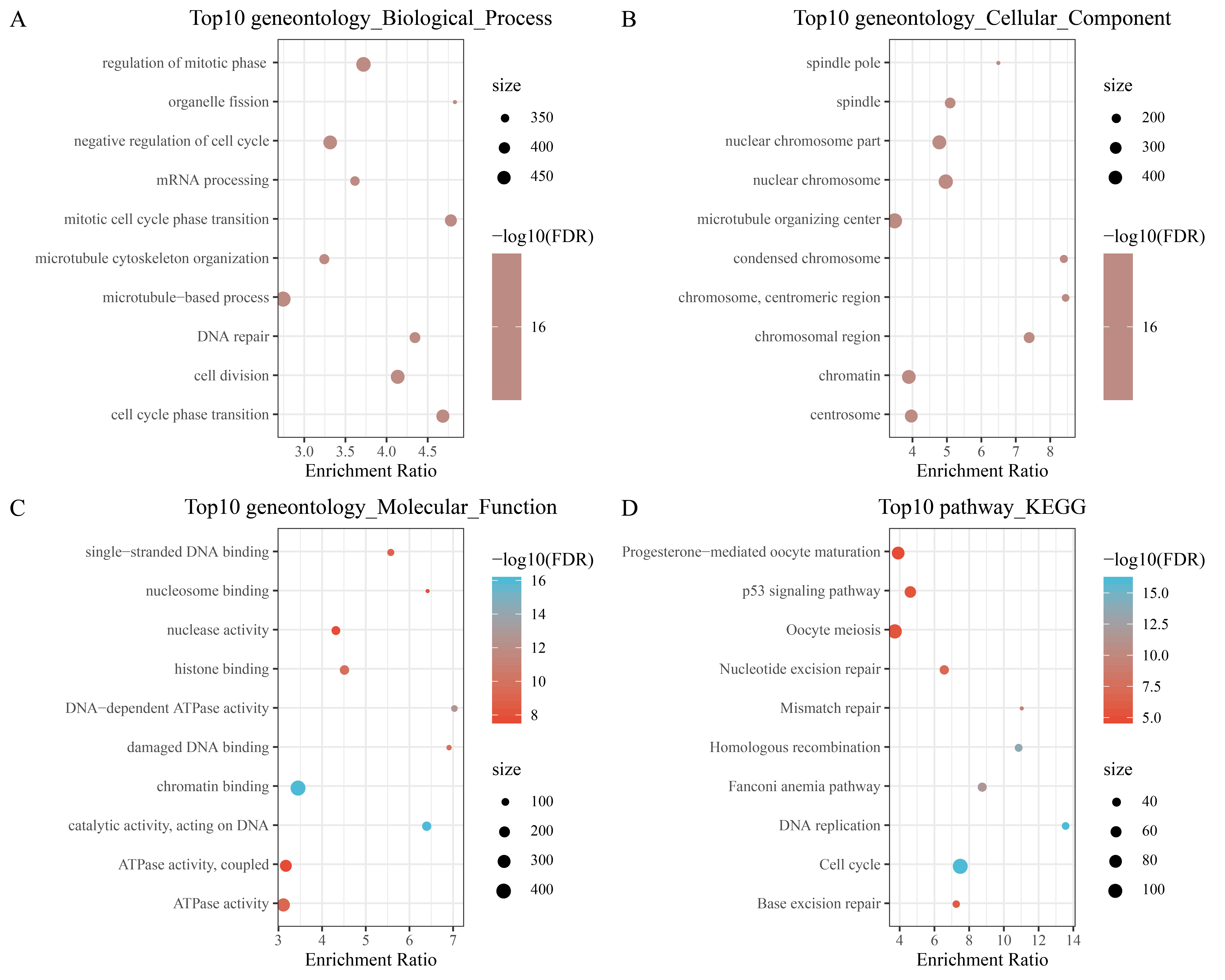

Supplement: Supplementary Figure 1 — GO and KEGG pathway enrichment analysis in the blue module. [file Image_1.TIF]

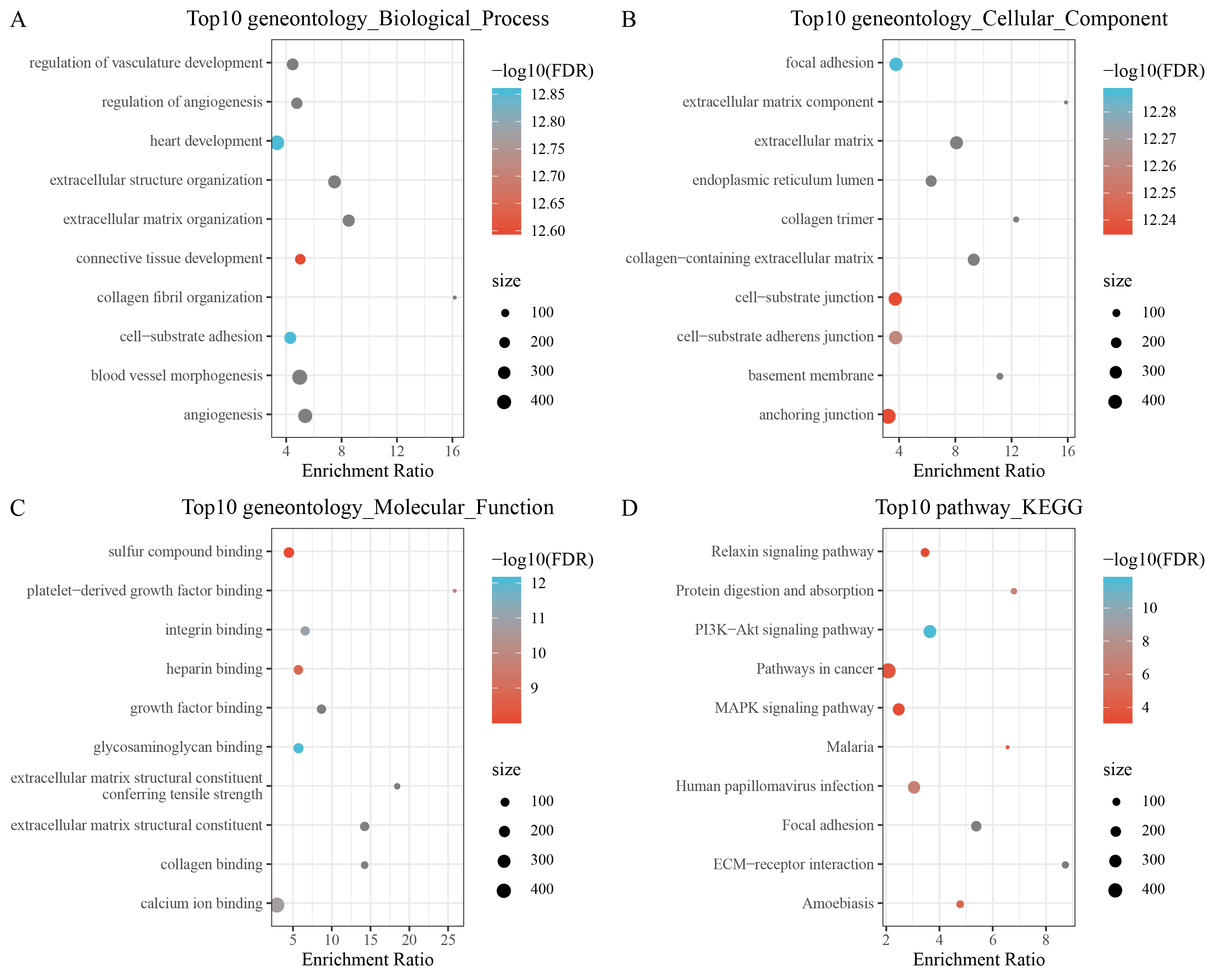

Supplement: Supplementary Figure 2 — GO and KEGG pathway enrichment analysis in the yellow module. [file Image_2.TIF]

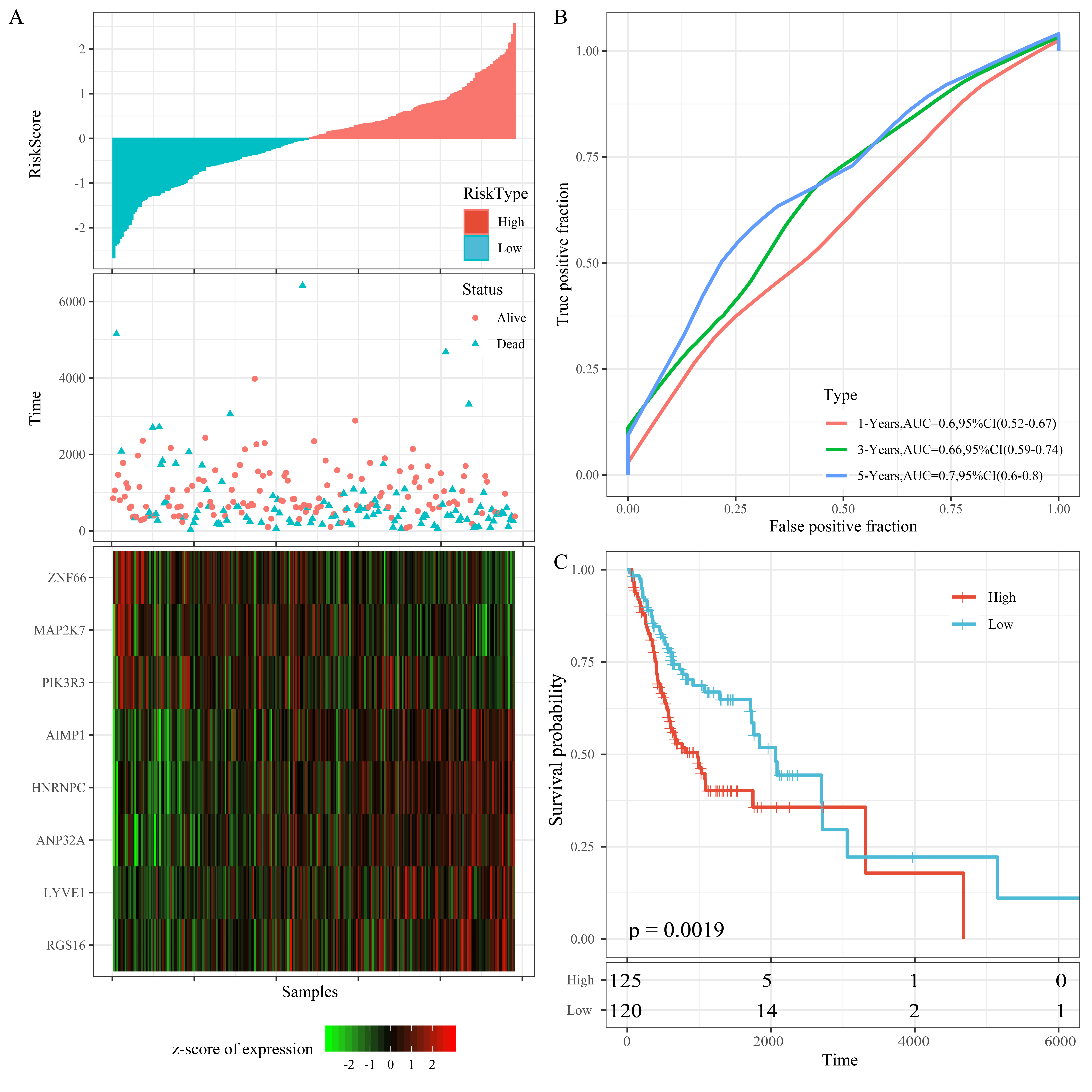

Supplement: Supplementary Figure 3 — Performance of the 8-mRNAsi based signature modelwith TCGA test dataset. [file Image_3.tif]

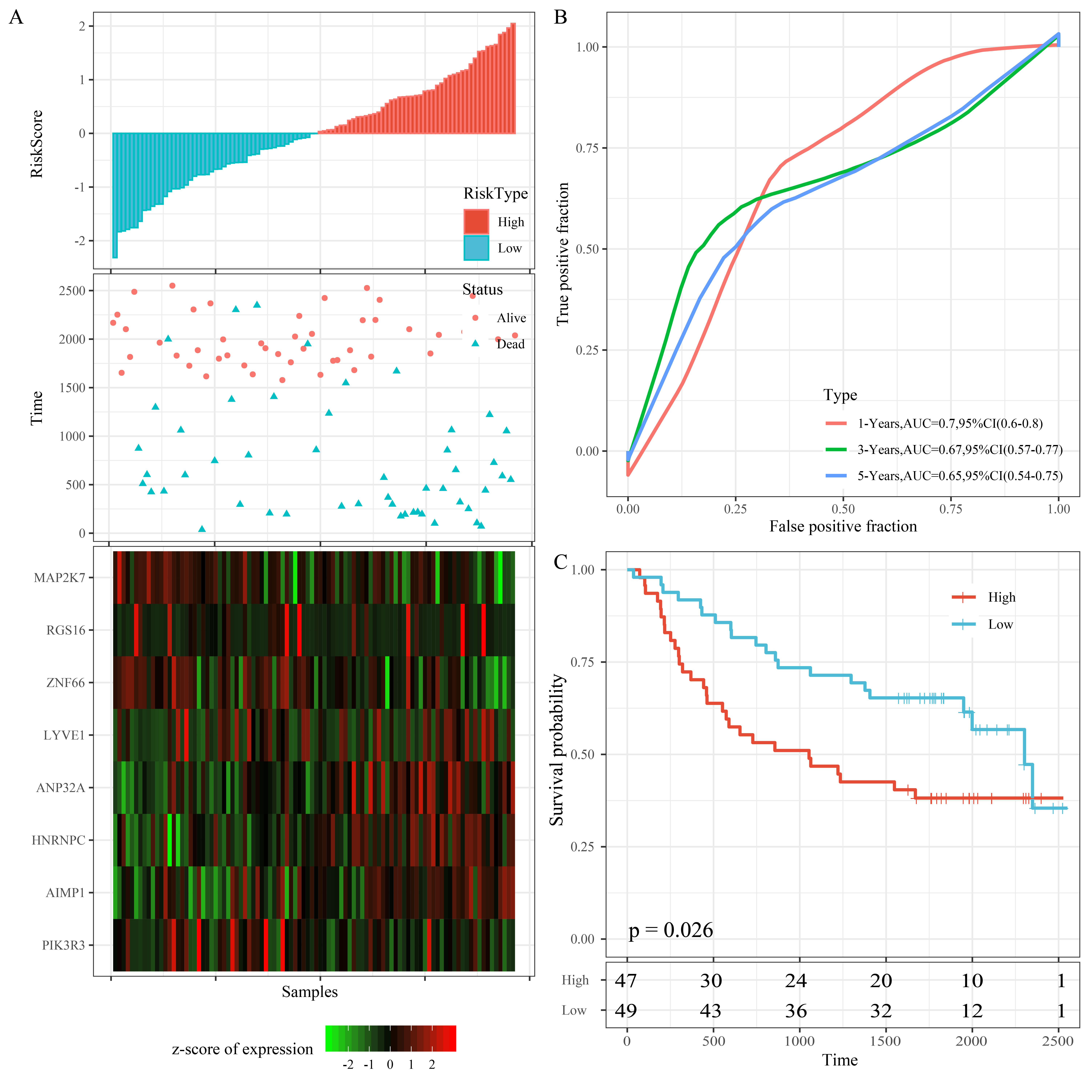

Supplement: Supplementary Figure 4 — Performance of the 8-mRNAsi based signature modelwithGSE41613 database. [file Image_4.tif]
