# Supplementary material for: Identifying 8-mRNAsi Based Signature for Predicting Survival in Patients With Head and Neck Squamous Cell Carcinoma via Machine Learning
Source: Front Genet. 2020 Oct 29;11:566159. doi: 10.3389/fgene.2020.566159 (PMC7721480; doi:10.3389/fgene.2020.566159)
Supplement: Supplementary Table 1 — The sequence of primers used for PCR in this study. [file Table_1.DOCX]

The sequence of primers used for PCR in this study

| RGS16 primer |
| --- |
| F：5’- GCTTCCTGAAGTCGCCTGCTTAC-3’ |
| R：5’- TTCCTCACTGCCGTGGAGACTC-3’ |
| LYVE1 primer |
| F：5’- GATTCCGCAGCTCAGACCCTTTC-3’ |
| R：5’- ACTGTCGTATCCTCAGCCTTGTTCTA-3’ |
| HNRNPC primer |
| F：5’- AGGCTGAAGTATGAACTACCCTTGGA-3’ |
| R：5’- CATCTGACACTCTCTCCTAAGCATCC-3’ |
| ANP32A primer |
| F：5’- CATTCCAGATGAACTGCTAGGTGAGG-3’ |
| R：5’- TGAAGATACACCACTGAGTCTTGCTT-3’ |
| AIMP1 primer |
| F：5’- ACAGCAGTAACAACCGTATCTTCTGG-3’ |
| R：5’- CTATTGGCTTAGAGTCGGCACTTCC-3’ |
| ZNF66 primer |
| F：5’- AGCCTTCAGTCGGTCCTCTATTCTT-3’ |
| R：5’- GCCTTGCCACATTCTTCACATTTGT-3’ |
| PIK3R3 primer |
| F：5’- ATCCTTGGTTCAGCACAACGACTC-3’ |
| R：5’- CCACCTCTCTTCCCACTTCCTCTT-3’ |
| MAP2K7 primer |
| F：5’- AGATGACAGTGGCGATTGTGAAGG-3’ |
| R：5’- GCAGGATGTTGGAGGGCTTGAC-3’ |
| GAPDH primer |
| F：5’- TCAAGAAGGTGGTGAAGCAGG-3’ |
| R：5’- GCGTCAAAGGTGGAGGAGTG-3’ |
